# Korean Red Ginseng Prevents the Deterioration of Lung and Brain Function in Chronic PM_2.5_-Exposed Mice by Regulating Systemic Inflammation

**DOI:** 10.3390/ijms241713266

**Published:** 2023-08-26

**Authors:** Ju Hui Kim, Jong Min Kim, Hyo Lim Lee, Min Ji Go, Tae Yoon Kim, Seung Gyum Joo, Han Su Lee, Ho Jin Heo

**Affiliations:** Division of Applied Life Science (BK21), Institute of Agriculture and Life Science, Gyeongsang National University, Jinju 52828, Republic of Korea; zkfkapflove@nate.com (J.H.K.); myrock201@gnu.ac.kr (J.M.K.); gyfla059@gnu.ac.kr (H.L.L.); rh9245@naver.com (M.J.G.); kty8747@gnu.ac.kr (T.Y.K.); s716g@naver.com (S.G.J.); ns3005@naver.com (H.S.L.)

**Keywords:** Korean red ginseng, *Panax ginseng*, particulate matter, cognitive dysfunction, lung fibrosis, inflammation

## Abstract

This study was conducted to confirm the effects of Korean red ginseng on lung and brain dysfunction in a BALB/c mice model exposed to particulate matter (PM)_2.5_ for 12 weeks. Learning and cognitive abilities were assessed with Y-maze, passive avoidance, and Morris water maze tests. To evaluate the ameliorating effect of red ginseng extract (RGE), the antioxidant system and mitochondrial function were investigated. The administration of RGE protected lung and brain impairment by regulating the antioxidant system and mitochondrial functions damaged by PM_2.5_-induced toxicity. Moreover, RGE prevented pulmonary fibrosis by regulating the transforming growth factor beta 1 (TGF-β1) pathway. RGE attenuated PM_2.5_-induced pulmonary and cognitive dysfunction by regulating systemic inflammation and apoptosis via the nuclear factor kappa-light-chain-enhancer of activated B cells (NF-κB)/c-Jun N-terminal kinases (JNK) pathway. In conclusion, RGE might be a potential material that can regulate chronic PM_2.5_-induced lung and brain cognitive dysfunction.

## 1. Introduction

Air pollution has natural and anthropogenic causes that affect human health and cause an increase in disease [1]. Particulate matter (PM) contains a variety of pollutants, including heavy metals, sulfates, nitrates, volatiles, and organic compounds [2]. PM_2.5_ is inhaled into the airway epithelial cells through respiration, affects mucociliary movement, is deposited in the alveoli, and interferes with the functioning of alveolar macrophages [3]. Chronic PM_2.5_ exposure induces the production of oxidative stress and reactive oxygen species (ROS), leading to an increase in pro-inflammatory cytokines [4]. Activation of cytokines induces cellular and tissue damage throughout the circulation, resulting in systemic inflammation and deterioration of lung and brain function [5]. Through respiratory and systemic circulation, PM_2.5_ produces inflammatory mediators such as transforming growth factor-beta (TGF-β) and tumor necrosis factor-alpha (TNF-α) in the lungs and extrapulmonary organs [6]. Furthermore, transition metals such as Fe, Mg, Al, and Cu in the PM damage the blood–brain barrier (BBB) by forming ROS and pro-inflammatory cytokines [7]. Soluble transition metals in PM that have passed through the BBB cause inflammatory responses and functional disorders in the brain [8]. Therefore, exposure to PM_2.5_ causes various health problems, including chronic respiratory and cardiovascular diseases, myocardial infarction, and asthma [9]. Most research is focused on the consequences of cardiovascular and respiratory diseases, but there are relatively few studies on the toxic effects of PM_2.5_ and systemic inflammation [10].

Korean red ginseng (*Panax ginseng*) is well known as a traditional medicinal herb in East Asia and has tonic properties that maintain or restore the physical condition to normal [11]. Korean red ginseng contains saponins such as ginsenosides and protopanaxatriol and non-saponin components such as pyroglutamic acid and p-coumaric acid and is known to have various physiological activities [12]. These physiological activities are known to enhance immune function and have anti-obesity, anti-diabetic, and anti-depressive effects [13]. In addition, Korean red ginseng regulates the nuclear factor kappa-light-chain-enhancer of activated B cells (NF-κB) pathway stimulated by lipopolysaccharide and inhibits the expression of transcription factors such as TNF-α and cyclooxygenase-2 (COX-2) [14]. However, there are few studies on lung function and brain cognitive dysfunction after long-term exposure to PM_2.5_. Therefore, we evaluated the effects of Korean red ginseng on lung and brain dysfunction in systemic inflammation proceeded by PM_2.5_ toxicity.

## 2. Results

### 2.1. Animal Behavioral Tests

**Figure 1:**
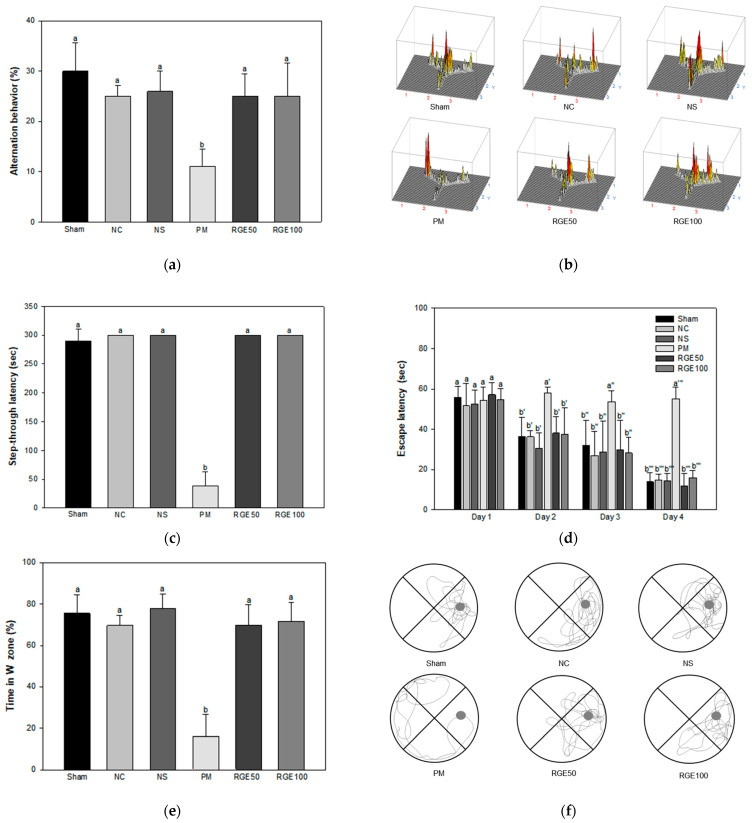
Protective effect of red ginseng extract (RGE) on PM_2.5_-induced mice. (**a**) alternation behavior; (**b**) 3D moving routes in the Y-maze test; (**c**) latency after learning in the passive avoidance test; (**d**) escape latency in the hidden trial; (**e**) time in the W zone; (**f**) routes tracking of the probe test in Morris Water Maze test. Different small letters suggest meaningful differences (*p* < 0.05). The results are exhibited as mean ± SD (*n* = 7).

In the results of the Y-maze test, there was no meaningful variation in the number of arm entries in the sham control group (Sham), normal control group (NC), normal sample group (NS), particulate matter group (PM), red ginseng extract 50 mg/kg of body weight group (RGE 50), and red ginseng extract 100 mg/kg of body weight group (RGE 100) (Figure S1). As a result of the alternation behavior tests, there was no meaningful difference among the Sham (29.98%), NC (25.05%), and NS (25.96%) groups. The PM group (11.03%) significantly declined spontaneous alternation behavior compared with the NC group. The RGE groups (RGE50, 24.99%; RGE100, 24.95%) increased more than the PM group (Figure 1a). As a result of tracing the movement path in the Y-maze test, RGE groups were similar to Sham, NC, and NS groups in arm entry movement and showed more active movement than the PM group (Figure 1b).

In the results of the passive avoidance test, the training session of latency during habitation indicated no meaningful difference among all groups (Figure S2). In the step-through latencies on the test session, there was no meaningful difference among the Sham (289.83 s), NC (300.00 s), and NS (300.00 s) groups. Those of the PM group (38.83 s) especially decreased more than the NC group. Whereas those of the RGE groups (RGE50, 300.00 s; RGE100, 300.00 s) increased more than the PM group (Figure 1c).

In the results of the Morris water maze test, on the first day of the training session, all groups indicated no meaningful difference (Figure 1d). On the last day of the training period, the escape latency of the PM group (55.29 s) increased considerably more than the Sham (13.91 s), NC (14.77 s), NS (14.56 s), RGE50 (11.99 s), and RGE100 (16.04 s) groups. In the probe test, the relative retention time in the W zone of the PM group (16.03%) declined more than those of the Sham (75.45%), NC (69.58%), and NS (77.72%) groups. On the other hand, those of the RGE groups (RGE 50, 69.92%; RGE100, 71.72%) increased more than the PM group (Figure 1e). As a result of tracing the movement path of the probe test in the Morris water maze test, the RGE groups showed movement around the W zone similar to Sham, NC, and NS groups and stayed in the W zone for a longer time than the PM group (Figure 1f).

### 2.2. Antioxidant System

**Figure 2:**
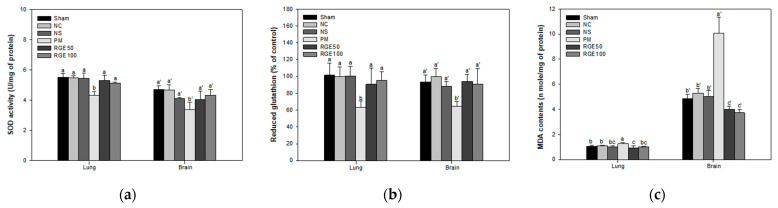
Protective effect of red ginseng extract (RGE) on PM_2.5_-induced mice. (**a**) SOD activity; (**b**) GSH contents; (**c**) MDA contents in lung and brain tissues. Different small letters a–c = lung and a’–c’ = brain suggest meaningful different tissues (*p* < 0.05). The results are exhibited as mean ± SD (*n* = 5).

The superoxide dismutase (SOD) activity in lung and brain tissue among the Sham (lung, 5.52 unit/mg of protein; brain, 4.70 unit/mg of protein), NC (lung, 5.49 unit/mg of protein; brain, 4.70 unit/mg of protein), and NS (lung, 5.46 unit/mg of protein; brain, 4.11 unit/mg of protein) groups indicated no considerable differences. However, lung tissue among the RGE groups (RGE 50, 5.33 unit/mg of protein; RGE100, 5.15 unit/mg of protein) and brain tissue among the RGE groups (RGE 50, 4.05 unit/mg of protein; RGE100, 4.33 unit/mg of protein) were substantially improved over the PM group (lung, 4.31 unit/mg of protein; brain, 3.37 unit/mg of protein) (Figure 2a).

The reduced glutathione (GSH) contents in lung and brain tissue among the Sham (lung, 101% of control; brain, 93.5% of control), NC (lung, 100% of control; brain, 100% of control), and NS (lung, 100% of control; brain, 88.0% of control) groups indicated no significant differences. However, lung tissue among the RGE groups (RGE 50, 90.6% of control; RGE100, 95.2% of control) and brain tissue among the RGE groups (RGE 50, 94.1% of control; RGE100, 90.8% of control) was significantly increased over the PM group (lung, 63.4% of control; brain, 64.7% of control) (Figure 2b).

The malondialdehyde (MDA) contents in lung and brain tissue among the Sham (lung, 1.06 nmole/mg of protein; brain, 4.85 nmole/mg of protein), NC (lung, 1.10 nmole/mg of protein; brain, 5.30 nmole/mg of protein), and NS (lung, 1.02 nmole/mg of protein; brain, 5.01 nmole/mg of protein) groups indicated no meaningful differences. However, lung tissue among the RGE groups (RGE 50, 0.95 nmole/mg of protein; RGE100, 1.03 nmole/mg of protein) and brain tissue among the RGE groups (RGE 50, 4.01 nmole/mg of protein; RGE100, 3.73 nmole/mg of protein) was substantially decreased compared to the PM group (lung, 1.27 nmole/mg of protein; brain, 10.1 nmole/mg of protein) (Figure 2c).

### 2.3. Mitochondrial Activity

**Figure 3:**
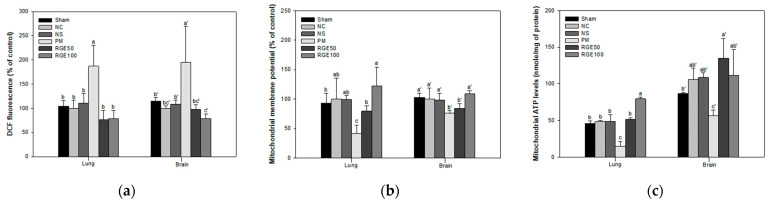
Protective effect of red ginseng extract (RGE) on PM_2.5_-induced mice. (**a**) Mitochondrial ROS activity; (**b**) MMP activity; (**c**) mitochondrial ATP levels in lung and brain tissues. Different small letters a–c = lung and a’–c’ = brain suggest meaningful different tissues (*p* < 0.05). The results are exhibited as mean ± SD (*n* = 5).

The mitochondrial ROS activity in lung and brain tissues among the Sham (lung, 104% of control; brain, 115% of control), NC (lung, 100% of control; brain, 100% of control), and NS (lung, 110% of control; brain, 109% of control) groups showed no significant differences. However, lung tissue among the RGE groups (RGE 50, 76.4% of control; RGE100, 78.9% of control) and brain tissue among the RGE groups (RGE 50, 98.1% of control; RGE100, 78.6% of control) was significantly decreased over the PM group (lung, 187% of control; brain, 195% of control) (Figure 3a).

The mitochondrial membrane potential (MMP) levels in the lung and brain tissues among the Sham (lung, 93.2% of control; brain, 102% of control), NC (lung, 100% of control; brain, 100% of control), and NS (lung, 99.0% of control; brain, 98.1% of control) groups showed no significant differences. However, lung tissue among the RGE groups (RGE 50, 79.2% of control; RGE100, 122% of control) and brain tissue among the RGE groups (RGE 50, 84.5% of control; RGE100, 108% of control) was significantly increased over the PM group (lung, 41.5% of control; brain, 76.6% of control) (Figure 3b).

The ATP contents in the lung and brain tissues among the Sham (lung, 45.6 nmole/mg of protein; brain, 86.8 nmole/mg of protein), NC (lung, 48.5 nmole/mg of protein; brain, 105 nmole/mg of protein), and NS (lung, 48.7 nmole/mg of protein; brain, 108 nmole/mg of protein) groups indicated no meaningful differences. However, lung tissue among the RGE groups (RGE 50, 51.8 nmole/mg of protein; RGE100, 79.4 nmole/mg of protein) and brain tissue among the RGE groups (RGE 50, 135 nmole/mg of protein; RGE100, 111 nmole/mg of protein) was substantially improved over the PM group (lung, 14.8 nmole/mg of protein; brain, 56.3 nmole/mg of protein) (Figure 3c).

### 2.4. Fibrosis Protein Expression in Lung Tissue

**Figure 4:**
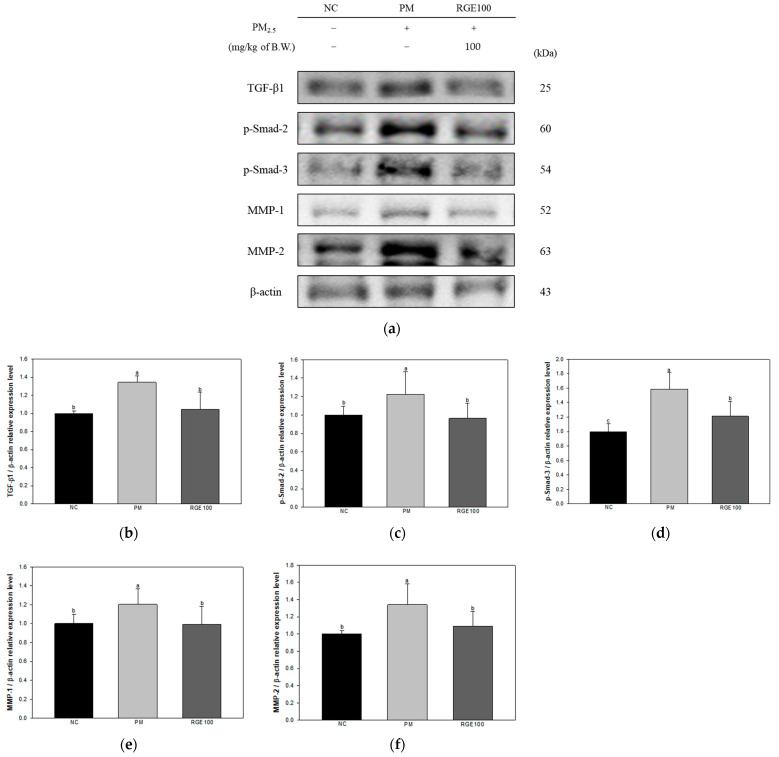
Protective effect of red ginseng extract (RGE) on PM_2.5_-induced mice. (**a**) Protein expression of Western blot image; (**b**) Protein expression levels of TGF-β1; (**c**) p-Smad-2; (**d**) p-Smad-3; (**e**) MMP-1; (**f**) MMP-2 in lung tissues. Different small letters suggest meaningful differences (*p* < 0.05). The results are exhibited as mean ± SD (*n* = 3).

The expression of proteins related to the fibrosis response in the lung tissue of mice exposed to PM_2.5_ is presented in Figure 4a. The expression levels of TGF-β1 (135%), phosphorylated-suppressor of mothers against decapentaplegic (p-Smad)-2 (123%), p-Smad-3 (159%), matrix metalloproteinase (MMP)-1 (120%), and MMP-2 (134%) in the PM group increased more than the NC group (100%). Whereas the expression levels of TGF-β1 (104%), p-Smad-2 (96.3%), p-Smad-3 (121%), MMP-1 (99.2%), and MMP-2 (109%) in the RGE100 group showed decreased expression levels compared to the PM group (Figure 4b–f).

### 2.5. Inflammatory and Apoptotic Protein Expression in Lung Tissue

**Figure 5:**
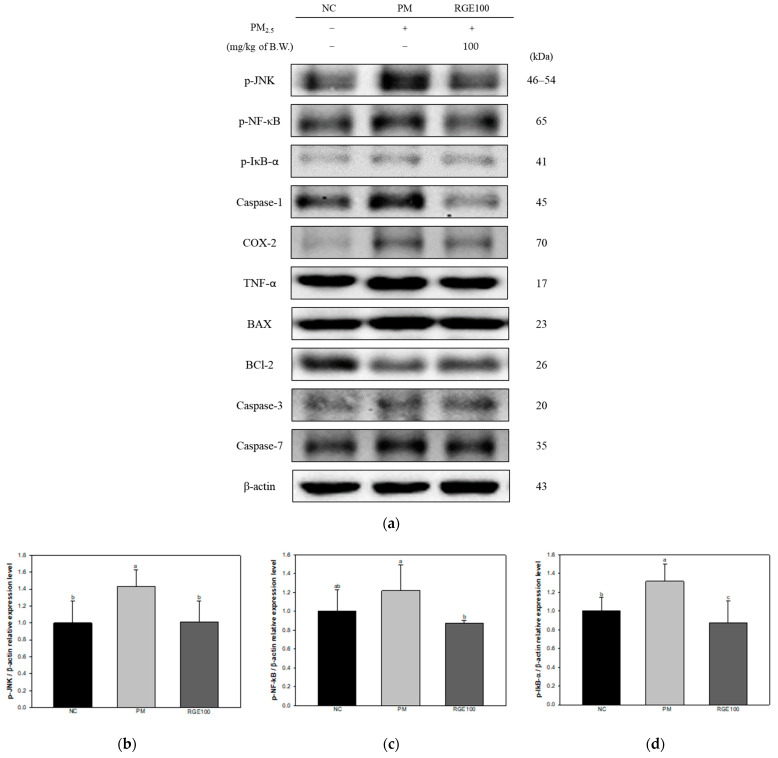

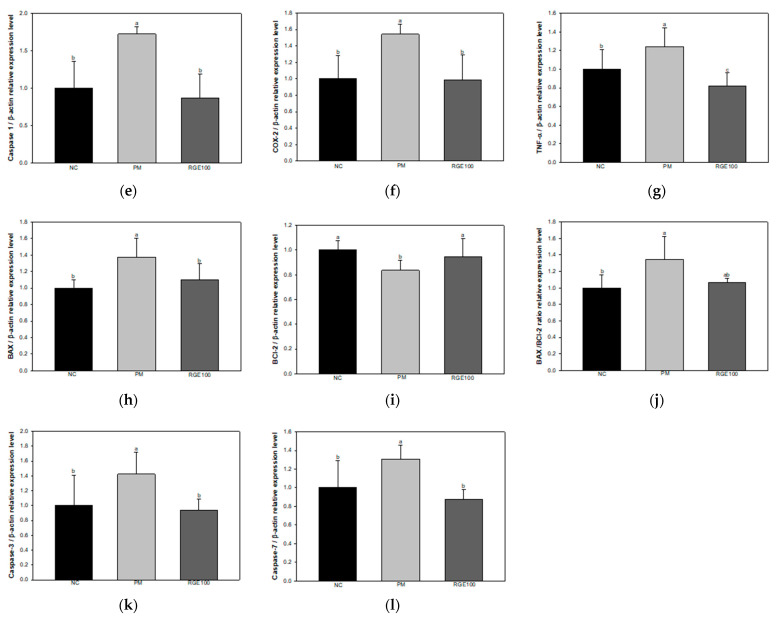
Protective effect of red ginseng extract (RGE) on PM_2.5_-induced mice. (**a**) Protein expression of Western blot image; (**b**) Protein expression levels of p-JNK; (**c**) p-NF-κB; (**d**) p-IκB-α; (**e**) Caspase-1; (**f**) COX-2; (**g**) TNF-α; (**h**) BAX; (**i**) BCl-2; (**j**) BAX/BCl-2 ratio; (**k**) Caspase-3; (**l**) Caspase-7 in lung tissues. Different small letters suggest meaningful differences (*p* < 0.05). The results are exhibited as mean ± SD (*n* = 3).

The expression of proteins related to the inflammatory and apoptotic response in the lung tissue of mice exposed to PM_2.5_ is presented in Figure 5a. The inflammatory protein expression levels of phosphorylated-c-Jun N-terminal kinases (p-JNK) (143%), phosphorylated nuclear factor kappa-light-chain-enhancer of activated B cells (p-NF-κB) (113%), phosphorylated nuclear factor of kappa light polypeptide gene enhancer in B-cells inhibitor, alpha (p-IκB-α) (131%), Caspase-1 (172%), COX-2 (154%), and TNF-α (123%) in the PM group increased more than the NC group (100%). Whereas the RGE100 group showed decreased p-JNK (101%), p-NF-κB (81.6%), p-IκB-α (87.4%), Caspase-1 (86.9%), COX-2 (99.0%), and TNF-α (82.2%) expression levels compared to the PM group (Figure 5b–g).

The apoptotic protein expression levels of B-cell lymphoma 2 (BCl-2)-associated X protein (BAX) (137%), Caspase-3 (142%), and Caspase-7 (130%) in the PM group increased more than the NC group (100%). Whereas the RGE100 group showed decreased BAX (109%), Caspase-3 (94.0%), and Caspase-7 (87.4%) expression levels compared to the PM group. BCl-2 (83.5%) expression levels in the PM group decreased more than in the NC group. The RGE100 group had increased BCl-2 (94.7%) expression levels compared to the PM group (Figure 5h–l).

### 2.6. Inflammatory and Apoptotic Protein Expression in Cerebral Cortex and Hippocampus Tissues

**Figure 6:**
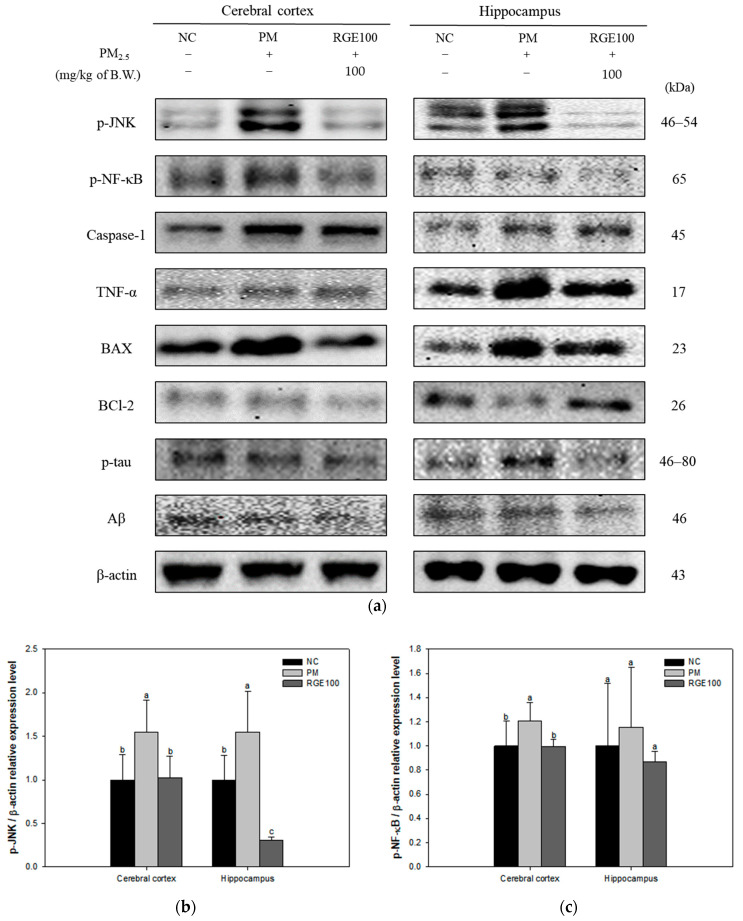

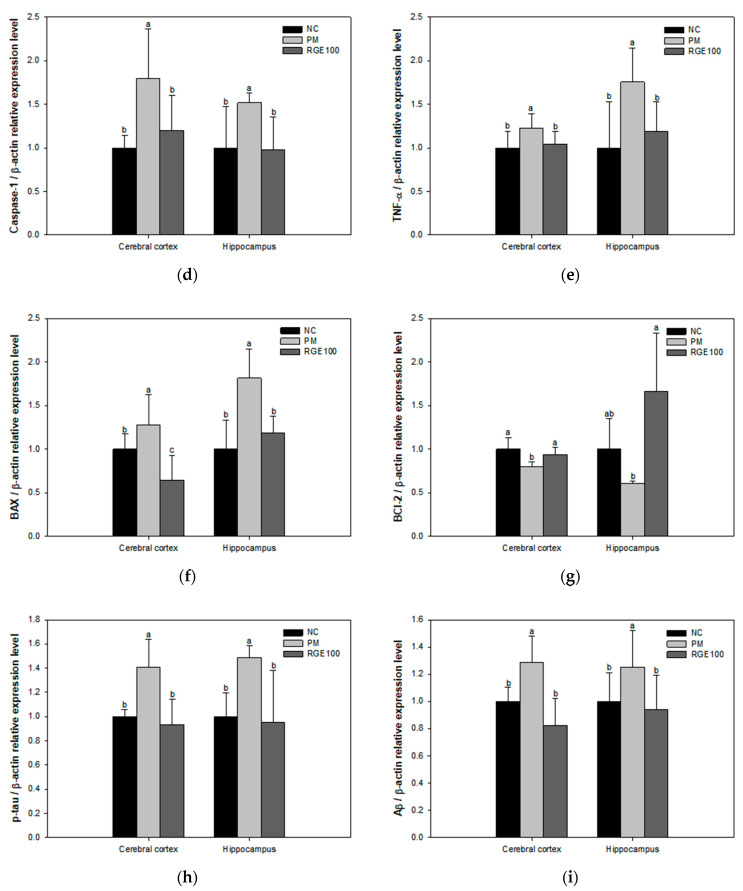
Protective effect of red ginseng extract (RGE) on PM_2.5_-induced mice. (**a**) Protein expression of Western blot image; (**b**) Protein expression levels of p-JNK; (**c**) p-NF-κB; (**d**) Caspase-1; (**e**) TNF-α; (**f**) BAX; (**g**) BCl-2; (**h**) p-tau; (**i**) Aβ in the cerebral cortex and hippocampus tissues. Different small letters suggest meaningful differences (*p* < 0.05). The results are exhibited as mean ± SD (*n* = 3).

The expression of proteins related to the inflammatory and apoptotic response in the cerebral cortex and hippocampus tissue of mice exposed to PM_2.5_ is presented in Figure 6a. The inflammatory protein expression levels of p-JNK (cerebral cortex, 154%; hippocampus, 155%), p-NF-κB (cerebral cortex, 120%; hippocampus, 171%), Caspase-1 (cerebral cortex, 179%; hippocampus, 151%), and TNF-α (cerebral cortex, 123%; hippocampus, 175%) in the PM group increased more than the NC group (100%). Whereas the expression levels of the RGE100 group were substantially downregulated for p-JNK (cerebral cortex, 102%; hippocampus, 30.9%), p-NF-κB (cerebral cortex, 99.6%; hippocampus, 104%), Caspase-1 (cerebral cortex, 120%; hippocampus, 98.0%), and TNF-α (cerebral cortex, 104%; hippocampus, 119%) compared to the PM group (Figure 6b–e).

The apoptotic protein expression levels of BAX (cerebral cortex, 127%; hippocampus, 181%), phosphorylated-tau (p-tau) (cerebral cortex, 140%; hippocampus, 148%), and amyloid β (Aβ) (cerebral cortex, 128%; hippocampus, 125%) in the PM group increased than the NC group (100%). Whereas the expression levels of the RGE100 group were significantly decreased for BAX (cerebral cortex, 64.3%; hippocampus, 118%), p-tau (cerebral cortex, 93.2%; hippocampus, 95.0%), and Aβ (cerebral cortex, 82.4%; hippocampus, 94.0%) compared to the PM group. BCl-2 (cerebral cortex, 80.1%; hippocampus, 61.1%) expression levels in the PM group decreased more than in the NC group. The RGE100 group increased BCl-2 (cerebral cortex, 93.8%; hippocampus, 166%) expression levels more than the PM group (Figure 6f–i).

## 3. Discussion

PM_2.5_ is an air pollutant that induces various health problems, and long-term exposure has been reported to increase mortality from cardiovascular disease, respiratory disease, stroke, and diabetes [15]. Therefore, in order to reduce the effect of PM on the human body, the WHO recently presented air quality guidelines to maintain the concentration of fine dust with a diameter of less than 2.5 µm at 25 µg/m^3^ per day for humans [16]. Because continuous exposure to these fine dust environments can easily lead to chronic disability and premature death, the WHO continuously updates air quality guidelines [17,18]. Inhaled PM_2.5_ triggers alveolar epithelial cell damage and induces inflammation and fibrosis in the lungs, and the pro-inflammatory mediators that are produced affect systemic circulation from the lungs to the brain [19]. PM_2.5_ absorbed through the BBB produces cytokines in the bloodstream, activating microglia, oxidative stress, and neuroinflammation in the brain [20]. Thus, this study was performed to evaluate the effect of red ginseng extract (RGE) on improving lung and brain tissue dysfunction on systemic inflammation from PM_2.5_-induced via the TGF-β1 and NF-κB/JNK pathways.

PM_2.5_ is deposited in the lungs, and some reach the brain through blood circulation, damages the hippocampus tissue responsible for memory and learning functions, and alters the function of neuronal synapses from the olfactory bulb to the brain, resulting in cognitive damage [6,21]. PM_2.5_ penetrates the BBB and induces pro-inflammatory cytokines, causing neuroinflammation and amyloid plaque formation [22]. In addition, an increase in brain inflammation and Aβ accumulation by exposure to PM_2.5_ ultimately causes Alzheimer’s disease (AD) [23]. In this study, behavioral tests were conducted to evaluate learning and memory abilities. PM_2.5_ caused cognitive dysfunction, and RGE prevented brain dysfunction by improving impaired short-term and long-term memory abilities (Figure 1). Ginsenosides from ginseng belonging to the genus *Panax* are triterpenoid saponins with a four-ring skeleton structure and are denatured under heat and acidic conditions [24]. During the manufacturing process of red ginseng, it is converted into ginsenosides such as Rg1, Rb1, Rb2, Rc, and Rd with heat treatment [25]. Ginsenosides from *Panax japonicus* inhibit oxidative stress and apoptosis and improve cognitive function by regulating Nrf2 and SIRT_1_ pathways related to neurodegenerative diseases in D-galactose-induced neuronal injury [26]. Administration of ginsenoside Rh1 and protopanaxatriol as ginsenoside Rg1 metabolites ameliorate scopolamine-induced cognitive decline by increasing learning and memory abilities and activating the hippocampus [27]. Ginsenosides Rg1 and Rb1 improve spatial learning ability by increasing the density of hippocampal synaptophysin, a synaptic protein involved in neurodevelopment, in Aβ-induced mice [28]. Ginsenoside Rh1 increases the survival rate of hippocampal cells and activates BDNF, which regulates nerve cells, confirming its potential as a therapeutic agent for neurodegenerative diseases [29]. Based on these results, RGE with various metabolites derived from ginsenosides with physiological activities might help improve PM_2.5_-induced cognitive decline.

Soluble transition metals or organic compounds on the surface of PM_2.5_ generate oxidative stress in various tissues [30]. Chronic exposure to PM_2.5_ increases the level of oxidative stress and damages the cellular antioxidant system, scavenging the production of ROS and oxidative radicals [31]. In addition, when the generated oxidative stress is not properly removed, the antioxidant system is compromised by causing an inflammatory response, apoptosis, and necrosis [32]. This damage to the antioxidant system reduces the activities of antioxidant enzymes such as SOD, GSH, and glutathione peroxidase and induces the production of lipid peroxidation and inflammatory cytokines in the lungs and brain [33]. Therefore, exposure to particulate matter reduces the conversion of oxidized glutathione disulfide (GSSG) to reduced GSH with the inhibition of glutathione reductase [34]. PM_2.5_ causes an inflammatory response by directly affecting the lungs, and PM_2.5_ penetrating brain tissue activates microglia and endothelial cells, causing neuroinflammation and neurodegeneration [35]. Similar to these results, exposure to PM_2.5_ increased oxidative stress and impaired antioxidant systems in lung and brain tissues. To evaluate the protective effect of RGE against oxidative stress caused by PM_2.5_ in the lung and brain, biomarker levels such as SOD, GSH, and MDA were confirmed. RGE increased the contents of SOD and GSH, which were reduced by PM_2.5_, and decreased the increased MDA contents (Figure 2). Ginsenoside Rg3 protects against cell and tissue damage from ROS generated by cyclophosphamide by enhanced SOD and catalase in the serum, thymus, and spleen [36]. In addition, ginsenoside Rh1 protects microglia damaged by H_2_O_2_ and inhibits ROS production, increasing antioxidant enzymes such as SOD-2 and catalase [37]. When *Panax ginseng* is consumed in diabetes induced by streptozotocin, it suppresses oxidative stress in the liver and increases the content of glutathione, showing antioxidant protection [38]. Based on these results, RGE inhibits ROS and oxidative stress generated by PM_2.5_, protects the antioxidant system, and attenuates lung and brain tissue damage.

Mitochondria are important organelles related to cellular functions as regulators of energy metabolism, apoptosis, and excessive oxidative stress [39]. However, exposure to PM_2.5_ causes inflammation and mitochondrial damage [40]. Transition metals and polyaromatic hydrocarbons in PM damage intracellular mitochondria, destroy the maintenance of membrane potential and calcium homeostasis and induce ROS production and apoptosis [41]. In addition, transition metals in PM induce ROS production by oxidizing organic compounds through the Fenton reaction [7]. ROS production reduces MMP levels and causes mitochondrial dysfunction. However, since MMP maintains intracellular energy homeostasis and suppresses mitochondrial metabolic disorders by increasing ATP synthesis, cell death must be prevented by increasing MMP levels [42]. In addition, ROS accumulation interferes with normal redox in tissues and shows toxic effects induced by PM_2.5_ with the production of free radicals and superoxide [31]. Similar to these studies, PM_2.5_ induced excessive ROS production in mitochondria and induced mitochondrial inactivation in lung and brain tissues. However, RGE improved mitochondrial function by suppressing ROS production and improving MMP levels that regulate apoptosis signaling and ATP levels that synthesize energy (Figure 3). Ginsenoside Rg1 regulates MMP, ATP, and ROS in cells damaged by Aβ toxicity and exerts a protective effect against mitochondrial dysfunction through cascade activation [43]. Ginsenoside Rd also protects mitochondrial mechanisms by regulating ROS generation, MMP degradation, and cytochrome C release induced by myocardial ischemia/reperfusion injury [44]. A recent study showed that ginsenoside Rg1 plays a neuroprotective role by increasing MMP and ATP levels and preventing Aβ-induced mitochondrial dysfunction [45]. RGE, containing various ginsenosides, improved mitochondrial function with the regulation of mitochondrial ROS content, MMP level, and ATP content and suppressed the production of inflammatory mediators induced by PM_2.5_.

PM_2.5_-induced inflammation and apoptosis in lung tissue activate lung fibrosis by inactivating alveolar macrophage function and causing mitochondrial DNA damage [46]. In damaged lung tissue, the production of cytokines increases, and autophagy is induced in cells by various mechanisms [47]. In addition, prolonged PM_2.5_ exposure stimulates the NLRP3 inflammasome activation/IL-1β/TGF-β1 signaling pathway to generate fibrosis [48]. TGF-β1 plays a role in regulating the damage and repair of lung tissue, which is activated by PM_2.5_ and inflammatory response, stimulates fibroblast proliferation and epithelial–mesenchymal transition, and promotes the expression of p-Smad family and MMPs activating lung fibrosis [49]. Activation of p-Smad2/Smad3 induces fibrosis by increasing fibroblasts [50]. In particular, MMP-1 and MMP-2 activate fibrosis and damage in lung tissue by inducing collagen deposition disorders and inhibiting pulmonary mitochondrial function [51]. Similar to these studies, PM_2.5_ exposure induced lung fibrosis by activating TGF-β1 and stimulated Smads and MMPs signals to induce lung tissue damage. However, RGE protected against lung fibrosis with TGF-β pathway regulation (Figure 4). *Panax ginseng* prevents lung tissue damage and fibrosis by regulating the TGF-β1/Smad and MMP pathways in bleomycin-induced pulmonary fibrosis [52]. Ginsenoside Rg1 prevented airway fibrosis and chronic lung disease by restricting the TGF-β1/Smad pathway [53]. In addition, ginsenoside Rg3 delayed the progression of pulmonary fibrosis by inhibiting hypoxia-inducible factor (HIF)/TGF-β1 pathway activation [54]. RGE, containing various ginsenosides with physiological activities, regulated pulmonary fibrosis caused by chronic PM_2.5_ exposure. By regulating the expression of factors involved in the TGF-β1/Smad pathway and the MMP pathway, it might be a useful material as a functional food to protect lung function.

PM_2.5_ entering the body activates macrophages and induces systemic inflammation, resulting in tissue dysfunction [9]. PM derived from the combustion of vehicle exhaust and fossil fuels destroys membrane stability and activates inflammatory responses and cytotoxic pathways [55]. Chronic PM_2.5_ exposure stimulates alveolar macrophages to produce cytokines and, through systemic circulation, stimulates key pathways in lung and brain dysfunction [56]. The inflammatory response of PM_2.5_ activates NF-κB, which increases the levels of inflammatory cytokines such as TNF-α, IL-1β, and COX-2, resulting in neurotoxicity and microglial activation [57,58]. In addition, exposure to PM_2.5_ induces oxidative stress that increases the number of macrophages in the lungs, and NF-κB/TNF-α pathway stimulation and Ca^2+^ influx result in cytochrome c release into the cytosol [59]. PM_2.5_ upregulated pro-apoptotic proteins such as caspase-3, caspase-9, and BAX and downregulated the anti-apoptotic protein BCl-2. It also released cytochrome c-induced caspases associated with apoptosis [60]. In particular, PM exposure impairs brain function with neuronal cell death and inflammatory responses and activates the amyloid beta and tau phosphorylation pathways, which are the cause of neurodegenerative diseases [61]. Amyloid plaques formed by amyloid aggregation are detected in the PM-damaged hippocampus and cerebral cortex and accelerate cytokines and inflammation in the brain. When the pathway is activated, Aβ and p-tau accumulate in the hippocampal region exposed to air pollutants, which can lead to neurodegenerative diseases [62]. In this study, PM_2.5_ showed protein expression of factors that regulate inflammatory and apoptotic responses in lung and brain tissues through the NF-κB/JNK pathway. However, RGE modulated PM._2.5_-induced cytokines suppressed signaling pathways and attenuated the inflammatory response (Figure 5 and Figure 6). Ginsenoside Rb1 attenuated and protected the TLR-2-mediated NF-kB and p-JNK pathways in *Staphylococcus aureus*-induced acute lung injury [63]. Ginsenoside Rg1 regulated the expression of the pro-inflammatory cytokines TNF-α and COX-2 in lung tissue by LPS-induced NF-κB activation [64]. Endothelial cells that regulate apoptosis in inflammation and angiogenesis are destroyed by cytokines and reactive oxygen species, but ginsenoside Rg3 inhibits apoptotic activity, regulates BAX and BCl-2, and inhibits caspase-3 and caspase-9 to protect them [65]. Ginsenoside Rg3 can induce apoptosis of lung cancer cells with the activation of the PINK1-Parkin signaling pathway, suggesting a protective role in mitophagy [66]. Additionally, ginsenoside Re improves diabetes-induced cognitive decline by inhibiting neuronal cell death by regulating JNK phosphorylation and p-tau aggregation in high-fat diet-induced diabetes [67]. Ginsenoside Rg3 improves inflammation and cytotoxicity by regulating the NF-kB pathway and inhibiting TNF-α expression in microglia against Aβ_42_ toxicity [68]. In Aβ_1–40_-induced Alzheimer’s disease (AD), ginsenoside Rb1 inhibits the expression of BAX and caspase-3 in the hippocampus and increases the expression of BCl-2, thereby regulating the neuronal cell death pathway and preventing AD [69]. Ginsenoside Rg1 prevents AD with its protective effects on memory capacity and neuronal synaptic plasticity in the hippocampus and inhibits the accumulation of amyloid beta and p-tau [70]. In conclusion, the toxicity of PM_2.5_ induces systemic inflammation with the regulation of ROS generation and cell signaling pathways. RGE improved lung and brain dysfunction with the regulation of the NF-κB/JNK pathway and prevented neurodegenerative diseases by protecting against brain neuroinflammation and apoptosis.

## 4. Materials and Methods

### 4.1. Preparation of Red Ginseng Extract (RGE)

RGE was provided by the Korea Ginseng Corporation (Buyeo, Republic of Korea) on 24 November 2021. Ginseng roots were washed and steamed by raising their temperature from 50 °C to 98 °C for 4 h. They were first dried for 15 h at 60~70 °C, and a secondary drying cycle was conducted in a closed chamber for 5 days at 50 °C. To prepare red ginseng extract, dried roots were consecutively extracted seven times for 12 h at 87 °C with distilled water. The filtered extracts were spray-dried to produce RGE and kept at −20 °C until use. The information on RGE’s ginsenoside compounds was supplied by Korea Ginseng Corporation (Daejeon, Republic of Korea) and is presented as follows (Rg1, 0.41 mg/g; Re, 0.46 mg/g; Rf, 1.50 mg/g; Rg2s, 2.01 mg/g; Rb1, 6.24 mg/g; Rc, 2.32 mg/g; Rb2 2.22 mg/g; Rd, 1.10 mg/g; Rg3s, 4.03 mg/g; Rg3r, 1.99 mg/g; Rh1, 1.55 mg/g). Red ginseng (*Panax ginseng*) is registered as a functional raw material for healthy functional foods in the Republic of Korea, and the standard for the content of functional ingredients is specified as more than 2.5 mg/g by combining Rg1, Rb1, and Rg3 [71].

### 4.2. Animal Experimental Design

**Figure 7:**
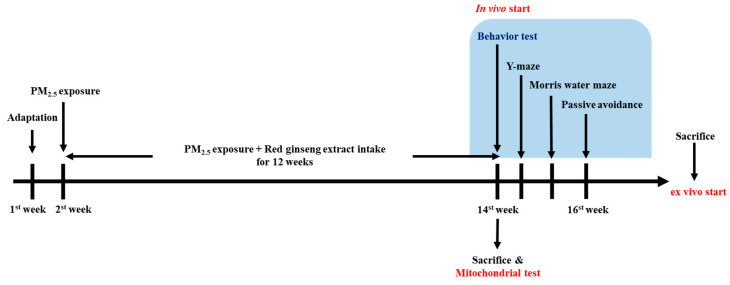
Experimental design of the animal behavioral test for PM_2.5_-induced mice.

BALB/c mice (male, 6 weeks) were obtained from Samtako (Osan, Republic of Korea). The mice were separated into four cages and maintained in a standard laboratory environment with a 22 ± 2 °C temperature, 12 h light/dark cycle, and 55% humidity. The animals were randomly separated into six groups: sham (without chamber exposure), normal control (clean air exposure), normal sample (clean air exposure + RGE 100 mg/kg of body weight), PM (PM_2.5_-exposed), RGE 50 (PM_2.5_-exposed + RGE 50 mg/kg of body weight) and RGE 100 (PM_2.5_-exposed + RGE 100 mg/kg of body weight). PM_2.5_ was bought from Power Technology Inc. (Arizona Test Dust, Arden Hills, MN, USA). The RGE was dissolved using clean drinking water and administered using a feeding needle one time a day before PM_2.5_ exposure. In this study, the concentrations of RGE fed to the mice were 50 and 100 mg/kg of body weight, and considering the corresponding intake for a 60 kg adult, the human equivalent doses (HED) are inferred to be 240 and 480 mg/60 kg/day [17]. The RGE concentration used in this experiment was set based on previous studies conducted on animals that could confirm the significant protective effect of Korean red ginseng [24,25,26]. Based on the evidence from Park et al. [72] that a dose between 40 and 200 mg/kg of body weight showed the best effect in mice exposed to fine dust, the study was conducted by setting it to between 50 and 100 mg/kg of body weight. The whole body of mice was continuously exposed to PM_2.5_ at a concentration of 500 μg/m^3^ [72]. PM_2.5_ was dissolved in filtered water and sprayed in aerosol form at an airflow rate of 10 L/min for 5 h/day for 12 weeks. All animal procedures were performed according to the procedures of the Animal Care and Use Committee of Gyeongsang National University (approval number: GNU-210803-M0069, approval day: 3 August 2021). Experimental protocols were performed following the Policy of the Ethical Committee of the Ministry of Health and Welfare, Republic of Korea. The experimental design is presented in Figure 7. After the behavioral tests, the mice were sacrificed using CO_2_ for ex vivo tests.

### 4.3. Animal Behavioral Tests

The Y-maze consists of a three-arm maze surrounded by walls measuring 33 cm long, 15 cm high, and 10 cm wide. The angle of each arm of the maze is the same. In the test, mice were placed at the end of one arm in the maze and allowed to move freely through the maze for 8 min. The percentage of alternating behaviors in which the mouse crossed the three arms was recorded. The mice’s motion was recorded using a Smart 3.0 video tracking system (Panlab, Barcelona, Spain). To conduct the Y-maze test, it was analyzed according to the method of Van der Borght et al. [73].

The passive avoidance chamber consists of two zones with a dark area that could shock electric shock and a lighted area divided by a central door. When the mice’s feet had moved completely and entered the dark part, an electric shock of 0.5 mA was applied for 3 s, and the step-through latency time of the lighted part was recorded. The passive avoidance test measurements were analyzed according to the method of Tsai et al. [74].

The Morris water maze consists of a circular pool 150 cm in diameter and 60 cm in height. The circular pool was filled with water and squid ink (Cebesa, Spain), and the temperature was maintained at 23 ± 2 °C. The test was separated into four quadrants labeled N, E, S, and W by marks on the outside of the pool. A black escape platform was placed in the center of the W zone so that it was not visible at the water level. Mice swam freely for 60 s until they found and climbed the platform, after which they were trained four times a day for 4 days in an environment where the platform was not visible 1 cm under the water’s surface. In the probe test, the mice swam freely in the platform-free pool for 60 s, and the retention time was recorded using a video tracking system (Smart v3.0, Panlab). To confirm the Morris water maze test, it was analyzed according to the method of Tsai et al. [74].

### 4.4. Antioxidant System

After behavioral tests, the lung and whole brain were homogenized with 10 × volume of phosphate-buffered saline (pH 7.4) using a bullet blender (Next Advance Inc., Averill Park, NY, USA). Experiments were conducted to measure antioxidant system biomarkers such as SOD, reduced GSH, and MDA contents. Protein concentration was determined by a microplate reader (Epoch 2, BioTek Instruments, Inc., Winooski, VT, USA) using the Bradford protein assay [75]. SOD content was estimated using a SOD kit (Sigma-Aldrich Chemical Co., St. Louis, MO, USA). Measurements of reduced GSH contents and MDA contents were performed according to previous studies [76].

### 4.5. Mitochondrial Activity

Lung and whole brain were homogenized by mixing mitochondrial isolation (MI) buffer that included 215 mM mannitol, 0.1% bovine serum albumin, 20 Mm 4-(2-hydroxyethyl)-1-piperazineethanesulfonic acid (HEPES) Na^+^, 75 mM sucrose and 1 mM ethylene glycol-bis(2-aminoethylether)-N, N, N′, N′-tetraacetic acid (EGTA) using a bullet blender. The mixture was centrifuged at 13,000× *g* at 4 °C for 10 min, and the pellet was combined with MI buffer containing 0.1% digitonin and MI buffer containing 1 mM EGTA. The combination was obtained by centrifuge (13,000× *g*, 4 °C for 15 min). The pellet was combined with MI buffer, and the mitochondrial activation was measured [77].

To measure the mitochondrial ROS content, the mitochondrial extract was resuspended with a respiration buffer (125 mM potassium chloride, 2 mM potassium phosphate monobasic, 2.5 mM malate, 20 mM HEPES, 1 mM magnesium chloride, 5 mM pyruvate, and 500 μM EGTA, pH 7.0) and 25 μM 2′,7′-dichlorofluorescein diacetate in a dark room for 20 min. The ROS contents were measured using a fluorometer microplate reader (Infinite F200, Tecan Co., San Jose, CA, USA) at 485 nm (excitation wave) and 535 nm (emission wave) [77].

To investigate the MMP level, the mitochondrial extract was reacted with 1 μM 5,5′,6,6′-Tetrachloro-1,1′,3,3′-tetraethylbenzimidazolylcarbocyanine iodide (JC-1) in MI buffer containing 5 mM pyruvate and 5 mM malate. The mixture was reacted in a dark room for 20 min, and fluorescence was measured at a wavelength of 530 nm (excitation filter) and 590 nm (emission filter) using a fluorometer microplate reader (Infinite F200) [77].

The mitochondrial ATP level was evaluated using an ATP assay kit (Sigma-Aldrich Chemical Co.) according to the manufacturer’s protocol. The ATP level was measured using a luminescence meter (GloMax-Multi+, Promega Corp., Madison, WI, USA) and calculated according to a standard curve.

### 4.6. Western Blot Assay

The lung, cerebral cortex, and hippocampal tissues were homogenized by mixing with ProtinExTM animal cell/tissue (Gene All Biotechnology, Seoul, Republic of Korea) including 1% protease inhibitor cocktails (Quartett, Berlin, Germany). The homogenates were centrifuged (13,000× *g*, 4 °C for 10 min). The supernatants were divided using sodium dodecyl sulfate-polyacrylamide gel and transferred to a polyvinylidene difluoride membrane (Millipore, Billerica, MA, USA). To block the membrane, it was incubated for 1 h with 5% skim milk, and the primary antibodies (1:1000) were reacted for 12 h at 4 °C. After that, the membranes were reacted with secondary antibodies (1:2000) for 1 h at room temperature. Primary antibodies and secondary antibodies for anti-mouse were bought from Cell Signaling Technology (Danvers, MA, USA) and Santa Cruz Biotechnology (Dallas, CA, USA). Finally, the chemiluminescence band was detected using an iBright CL 1000 Imaging System (Thermo Fischer Scientific, Waltham, MA, USA) [78].

### 4.7. Statistical Analysis

All results were expressed as the mean ± standard deviation (SD). Significant differences among the groups were established by one-way analysis of variation with Duncan’s multiple range test (*p* < 0.05) with the SAS software (version 9.4, SAS Institute Inc., Cary, NC, USA).

## 5. Conclusions

PM_2.5_ is a serious problem and induces a systemic inflammatory response. However, there are few drugs or foods that can effectively ameliorate the toxicity of PM_2.5_. In this study, chronically exposed PM_2.5_ is involved in the regulation of systemic inflammation. However, the administration of RGE improves PM_2.5_-induced cognitive dysfunction by regulating the damaged antioxidant system and mitochondrial activation. RGE regulated pulmonary fibrosis with the regulation of the TGF-β1 pathway. In addition, RGE protected the lung, cerebral cortex, and hippocampal tissues by regulating the NF-κB/JNK pathway involved in inflammatory responses and apoptosis signals. In conclusion, these results suggest that RGE might be used as a functional food material to improve lung and brain dysfunction with the control of systemic inflammation in chronic PM_2.5_-exposed mice.

## Data Availability

The data presented in this study are available on request from the corresponding author applicable.

## Supplementary Materials

The supporting information can be downloaded at: https://www.mdpi.com/article/10.3390/ijms241713266/s1.
