# Supplementary material for: Korean Red Ginseng Prevents the Deterioration of Lung and Brain Function in Chronic PM2.5-Exposed Mice by Regulating Systemic Inflammation
Source: Int J Mol Sci. 2023 Aug 26;24(17):13266. doi: 10.3390/ijms241713266 (PMC10488300; doi:10.3390/ijms241713266)
Supplement: Supplementary file 1 [file ijms-24-13266-s001.zip › ijms-2540442-supplementary.pdf]

Supplementary data

Figure S1. Protective effect of red ginseng extract (RGE) on $PM_{2.5}$-induced mice. Alternation behavior in Y-maze test. Different small letters suggest meaningful differences ($p < 0.05$). The results are exhibited as mean ± SD ($n = 7$).

Figure S2. Protective effect of red ginseng extract (RGE) on PM2.5-induced mice. Escape latency in hidden trial in Morris Water Maze test. Different small letters suggest meaningful differences ($p < 0.05$). The results are exhibited as mean ± SD ($n = 7$).

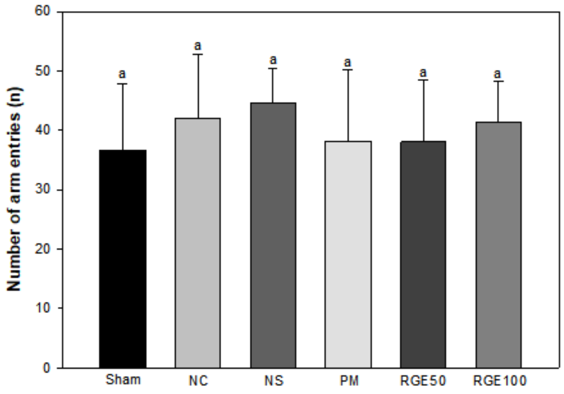

Figure S1

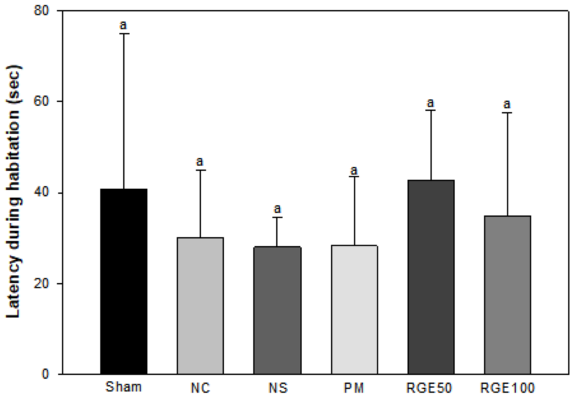

Figure S2
